# Application of McCoy Cell Line for Propagation of Herpes Simplex Virus Type 1

**Published:** 2015-05

**Authors:** Maryam Sadat Nabavinia, Sina Rostami, Faezeh Ghasemi, Zahra Meshkat

**Affiliations:** 1 Department of Biotechnology, School of Pharmacy, Mashhad University of Medical Sciences, Mashhad, Iran;; 2 Department of Biology, Faculty of Sciences, Ferdowsi University of Mashhad, Mashhad, Iran;; 3 Department of New Sciences and Technology, Faculty of Medicine, Mashhad University of Medical Sciences, Mashhad, Iran;; 4 Antimicrobial Resistance Research Center, Mashhad University of Medical Sciences, Mashhad, Iran

**Keywords:** Cell line, Herpes simplex virus type 1, Cytopathic effects, Virus titration

## Abstract

Herpes simplex virus types 1 (HSV-1) and 2 (HSV-2) are members of the *Herpesviridae* family. About 40% to 80% of the world populations are infected with HSV and its prevalence is high in Iran. The high prevalence of this virus in the community and the ability of the virus in causing fatal diseases among immunocompromised patients, have encouraged studies to be performed on HSV and suitable cell lines which supports the propagation of HSV. The aim of this study was to evaluate the suitability of McCoy cell line in the isolation and propagation of HSV. An isolated wild-type HSV-1 was obtained from the labial vesicles of a 29-year-old patient who was referred to Ghaem Hospital (Mashhad, Iran). The virus was inoculated in McCoy cell monolayer cells and its titer was calculated by 50% tissue culture infectious dose (TCID50) method. Cytopathic effects (CPE) of HSV on McCoy cells appeared about 20 hours after the infection of cells. Titer of the virus was 10^-5.25^ TCID50/ml. Our data showed that the McCoy cell line supported the propagation of HSV in high titer. This was the first study that used McCoy cell line for the isolation and propagation of HSV-1. McCoy cell line could be used, as a proper cell line of HSV, for various studies in the future.

## Introduction

Herpes simplex virus types 1 (HSV-1) and 2 (HSV-2) are double-stranded DNA viruses and are members of the *Herpesviridae* family. This family includes three subfamilies: alpha-, beta-, and gamma-herpesvirinae.^1^ HSV-1 and HSV-2 are capable of causing various diseases, including oral and genital lesions, blindness and encephalitis.^1^ Once infected with the virus, it may lead to viral latency in peripheral nerve ganglia.^2^

Unlike other members of the *Herpesviridae* family, HSV has low specificity to the host; therefore, they have the ability to infect a wide range of hosts. In addition to human cells, herpes viruses could infect animal cells. This feature has led to the successful spread of the viruses throughout the world. It is estimated that the virus has infected 40% to 80% of the world population.^3^

The prevalence of HSV is high in Iran. Studies on pregnant women in Tehran (Iran) showed that the level of neutralizing antibodies against HSV-1 and HSV-2 were 90.75% and 8.25%, respectively.^4^ In a study conducted on women in Shiraz, Iran, HSV-2 neutralizing antibodies were found in 28.19% of the studied patients.^5^ Studies have shown that HSV-2 infection increases the risk of HIV infection. As a result, development of a new vaccine against HSV infection may also serve in a better controlling the spread of HIV infection.^6^ The mechanism of cellular and molecular immunity against recurrent HSV infection is unknown; therefore, studies have been carried out in order to reveal the exact mechanism and develop an effective vaccine.^7^

Although the frequency of drug resistance to HSV is rare in immunocompetent people, it is common in immunocompromised patients and approximately 5% of their isolated viruses are resistant to antiviral drugs; however, this figure is higher (14-30%) in patients receiving bone marrow transplantation.^8^

Mathematical models have shown that anti-viral drugs for the treatment of HSV-2 may cause a gradual increase in drug resistant cases. Therefore, administration of antiviral drugs could not reduce the prevalence of this virus.^2^

The high prevalence of HSV in the community, its ability to be latent in infected individuals during their lifetime, and the ability of the virus for causing fatal diseases in immunocompromised patients highlights the increasing need to set up studies to develop vaccines and antiviral drugs. Designing suitable cell lines for the propagation of HSV is required for such studies. McCOY cell line was derived from human synovial fluid in1957 by Pomerat, but it changed between different laboratories for many years. Nogueira et al. demonstrated that this cell line has human and mouse cell’s marker. McCOY cell line is widely used by laboratories for the cultivation of various microorganisms.^9^

The aim of this study was to evaluate the ability of McCoy cell line for the isolation and propagation of HSV. 

## Patients and Methods

*Virus Isolation*

A wild type of HSV-1 was isolated from labial vesicles of a 29-year-old Iranian patient in 2010 and was identified as HSV-1 by PCR using HSV-1 specific primers. 

*Cell line and Virus Propagation*

McCoy cell line was purchased from Pasteur Institute (Iran). The virus was inoculated in McCoy cell monolayer in Dulbecco’s Modified Eagle Medium (DMEM) plus 5% heat inactivated fetal calf serum (FCS), 100 IU/ml penicillin and 100 μg/ml streptomycin at 37ºC with an atmosphere of 5% CO2. After the appearance of CPEs in more than 75% of the infected cells, the supernatant of the infected cells containing propagated viruses were collected and stored at 4ºC for one day.

*Virus titration*

1/log serial dilutions (10^-1^-10^-7^) of the stored virus seed were prepared in serum-free DMEM. Its titer was determined by 50% tissue culture infectious dose (TCID_50_) method as described previously.^10^ Tissue culture infectious dose 50% (TCID_50_) assay is a method used to quantitate infectious virus. This method is useful to determine titer of virus that cause a change in cellular morphology. Serial dilution of virus suspensions should provide in-tissue culture maintenance or growth medium to define virus titers and CPE observed different days after qualifying negative and positive control. TCID_50_ is determined by two methods, namely Reed-Muench and Spearman-Kärber. In this study Spearman-Kärber method was used to determine TCID_50_.

$$TCID50\;unit\;volume-1=Highest\;dilution\;giving\;100\%\;CPE+\frac{1}{2}-\frac{total\;number\;of\;test\;units\;showing\;CPE}{number\;of\;test\;units\;per\;dilution}$$

TCID_50_ ml^-1^=10*TCID_50_ unit volume^-1^.^11^

## Results

**Figure 1:**
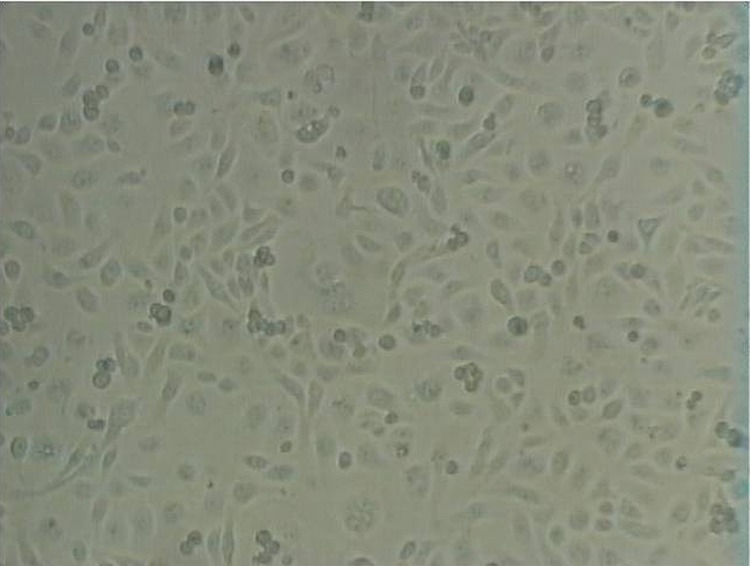
McCoy cells purchased from Pasteur Institute (Iran). Cells are adherent and have fibroblastic appearance.

Figure 1 shows McCoy monolayer cells. There is not enough information with regard to the origin of these cells in previous studies. Firstly, these cells were isolated from synovial fluid of the knee joint of a patient with degenerative arthritis, however, another sub-line was of mouse origin. These cells are adherent and have fibroblastic appearance. Cytopathic effects of HSV-1 on McCoy cells appeared about 20 hours after the infection of the cells.

**Figure 2:**
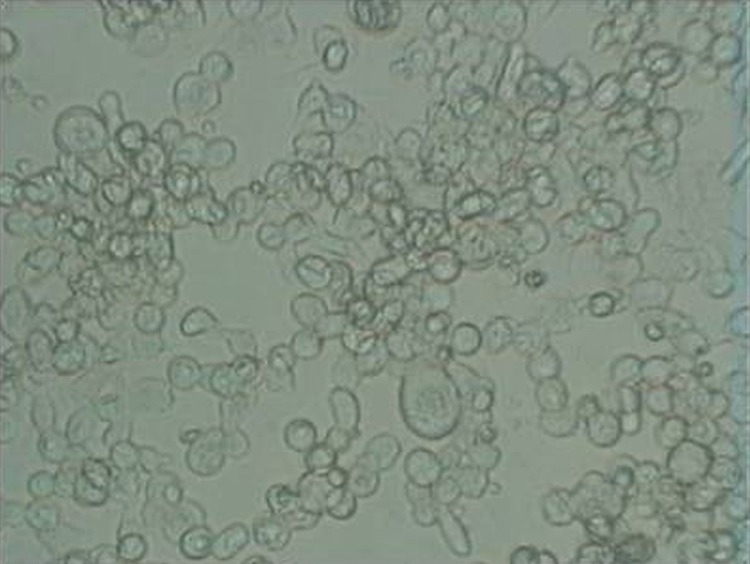
CPE of HSV-1 on the McCoy cell line. The CPE includes ballooning and clustering of infected cells and formation of multinucleated giant cells.

As shown in figure 2, HSV-1 CPEs includes ballooning and clustering of infected cells and formation of multinucleated giant cells.

TCID50 was calculated based on the ratio of the CPE positive wells to CPE negative wells (table 1) as described previously,^11^ and titer of the propagated virus was 10^-5.25^ TCID50/unit volume^-1^.

**Table 1:** Results of virus titration (TCID50 method) in McCoy cells

**Virus dilution**	**Number of inoculated wells**	**Number of positive Wells for CPE**	**CPE+wells/total numbers of wells**
10^-1^	4	4	1
10^-2^	4	4	1
10^-3^	4	4	1
10^-4^	4	4	1
10^-5^	4	3	0.75
10^-6^	4	0	0
10^-7^	4	0	0

## Discussion

Several cell lines, such as African green monkey kidney (Vero, CV-1), baby hamster kidney (BHK), fetal rhesus monkey kidney (FRhK), human embryonic lung (HEL), human fetal foreskin (ff), mink lung (ML), primary rabbit kidney (PRK), permanent rabbit kidney (RK13), and HeLa cell line have been used for HSV propagation ^12^^,^^13^. Among the mentioned cell lines, HEL and Vero cell lines showed the highest efficacy for the propagation of HSV.^12^ However, Sreedharan Athmanathan and his colleague showed immortalized human corneal epithelial cell line (HCE) was more susceptible compared with Vero cell line and the isolation of HSV-1 in clinical specimens by HCE was premiere to Vero cell line^14^ as HSV detection in cell culture is gold standard method but different cell line and cell culture method show distinct sensitivity.^15^ Finding more sensitive cell line could improve this method.

McCoy cells have been used in laboratories for about 50 years and are applied extensively for different studies. This cell line can be used for the culture of *Chlamydia*, studies for the design of vaccines, and as a cellular model for the studies of *Chlamydia trachomatis* cytotoxicity.^9^

The results of this study showed the high titer of isolated viruses. McCoy cells were used for the first time for the propagation of HSV in our study and high titers of propagated viruses were obtained. Our data showed that the McCoy cells could be used as a suitable cell line for HSV propagation. The clear CPE of the virus on infected cells are important for the detection of the virus. Although it is determined that the mechanism of HSV-1 dependent apoptosis in HEp-2/HeLa cells differ from Vero cells,^16^ further studies are required to determine the specific cellular receptors for HSV attachment and absorption and HSV-1 dependent apoptosis. McCoy-Plovdiv is the new generation of McCoy cell line that does not require serum for multiplication. The study of the ability of these cells for HSV propagation has not yet been determined. This cell line may prove useful for serum-free virus cultivation in studies such as vaccine development.^9^

## Conclusion

McCoy cell line can support the propagation of HSV-1. High titers of propagated virus could be obtained by culturing of HSV-1 in this cell line. 
